# Activity of catechins and their applications

**DOI:** 10.1186/s41702-020-0057-8

**Published:** 2020-02-26

**Authors:** Joonseo Bae, Nayoung Kim, Yunyoung Shin, Soo-Yeon Kim, You-Jeong Kim

**Affiliations:** 1 Department of Cosmetics Engineering, Graduate School of Konkuk University, 120 Neungdong-ro, Gwangjin-gu, Seoul, 05029 Republic of Korea; 2 Department of Beauty Art, Doowon Technical University, 159 Jurawui-gil, Paju-eup, Paju-si, Gyeonggi-do 10838 Republic of Korea; 3 Department of Beauty Yakson Care, Yeoju Institute of Technology, 338 Sejong-ro, Yeoju-si, Gyeonggi-do 12652 Republic of Korea

**Keywords:** Catechins, Activity, Anti-oxidant, Cosmetics, Ingredient

## Abstract

**Background:**

Catechins, which are polyphenol compounds found in many plants and are an important component of tea leaves, are strong anti-oxidants.

**Research:**

Many studies seek to enhance the effects of catechins on the human body and boost their protective power against UV radiation. There are many examples of the positive anti-microbial, anti-viral, anti-inflammatory, anti-allergenic, and anti-cancer effects of catechins. Catechins increase the penetration and absorption of healthy functional foods and bio cosmetics into the body and the skin, thus improving their utility. High value-added anti-oxidant substances have been extracted from food and plant sludge, and experiments have shown that catechins are safe when applied to the human body. The stability of catechins is very important for their absorption into the human body and the effectiveness of their anti-oxidant properties.

**Conclusion:**

Continued research on the strong anti-oxidant effects of catechins is expected to result in many advances in the food, cosmetics, and pharmaceutical industries.

## Background

**Fig. 1:**
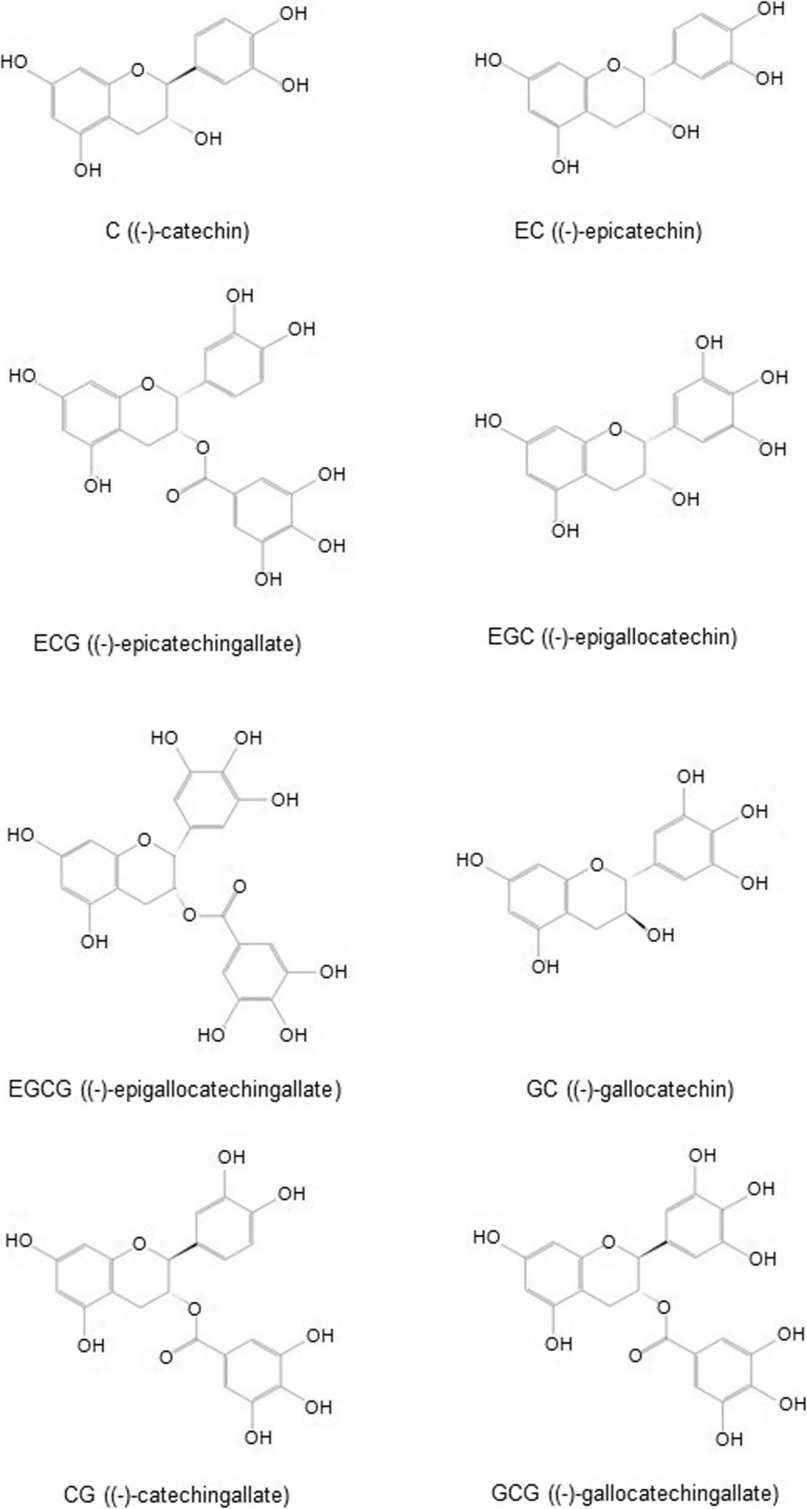
Structural formula of eight catechins. Catechins have many chemical structural features, such as hydroxyl groups (–OH), that combine easily with other materials. There are eight catechins: C ((-)-catechin), EC ((-)-epicatechin), ECG ((-)-epicatechingallate), EGC ((-)-epigallocatechin), EGCG ((-)-epigallocatechin gallate), GC ((-)-gallocatechin), CG ((-)-catechingallate), and GCG ((-)-gallocatechingallate). The principle types are C, EC, ECG, EGC, and EGCG

Catechins have many benefits including preventing or reducing skin damage. Catechins are important ingredients from tea leaves and have intensive anti-oxidant and representative physiological activities. They are members of the group of polyphenol compounds found in many medicinal plants. The major sources of catechins are *Camellia sinensis* (*C. sinensis*) and *C. assumica.* Green tea contains 75–80% water and polyphenol compounds (flavanols, flavandiols, flavonoid, and phenolic acid) (Zillich et al. 2015), and catechins account for more than 75% of the polyphenol compounds in tea leaves. They are condensation-type tannins with a ring and the basic structure of flavan-3-ol. They have many chemical structural features, such as hydroxyl groups (−OH), that combine easily with other materials (Singh et al. 2011). There are eight catechins (Fig. 1): C ((-)-catechin), EC ((-)-epicatechin), ECG ((-)-epicatechingallate), EGC ((-)-epigallocatechin), EGCG ((-)-epigallocatechin gallate), GC ((-)-gallocatechin), CG ((-)-catechingallate), and GCG ((-)-gallocatechingallate). The principle types are EC, ECG, EGC, and EGCG (Jin et al. 2006), which are prominently present in green tea (Fung et al. 2012). Catechins provide several health advantages by scavenging free radicals and retarding extracellular matrix degradation induced by ultraviolet (UV) radiation and pollution (Shi et al. 2016). Catechins also directly affect the skin by activating collagen synthesis and inhibiting the production of matrix metalloproteinase enzymes (Arct et al. 2003). Because of the hydroxyl in the gallate group, EGCG and ECG are highly effective free-radical scavengers compared with many other standard anti-oxidants, such as ascorbic acid, tocopherol, and trolox (Gulati et al. 2009; Matsubara et al. 2013; Kim et al. 2018). Because of these useful actions, tea catechins are increasingly used in medical, pharmaceutical, and cosmetic products and are being actively studied in a variety of approaches.

### Anti-oxidant activity

Catechins are well-studied substances with proven anti-oxidant effects. Studies have been conducted to boost the stability of catechins and increase their rate of absorption into the human body. Recent studies have focused on maximising the efficacy of anti-oxidants. Gallic acid and catechins show stable anti-oxidant activity by synthesis of galactan, and catechin anti-oxidants covalently bind to chains of proteins (Spizzirri et al. 2009). *Caesalpinia decapetala* (*C. decapetala*) is effective in the oxidation stability of an oil-in-water emulsion (Gallego et al. 2017). Analysis using LC-ESI/LTQ Orbitrap/MS of autochthonous germplasm of the Campania region showed a higher level of anti-oxidant activity compared with the non-autochthonous germplasm (D’Urso et al. 2018). Enzymatic glucosylation of caffeic acid and EGCG leads to improved anti-oxidant ability in a cellular model of UV-induced skin ageing (Nadim et al. 2014). The flamboyant tree (*Delonix regia*) has potent anti-oxidant and anti-microbial activities (Feng et al. 2014). EGCG anti-oxidant capacity is effective against H_2_O_2_-induced human dermal fibroblast injury (Feng et al. 2013). Lipophilized EGCG derivatives show increased anti-oxidant activity (Zhong and Shahidi 2011). Flavonoids and triterpenoids from the fruit of *Alphitonia neocaledonica* have cytotoxicity, anti-oxidant, and anti-tyrosinase activities and are useful cosmetic ingredients (Muhammad et al. 2014). Approximately 106 phenolic compounds have been found using liquid chromatography assays coupled with electrospray ionisation for rapid profiling of phenolic compounds from red maple (*Acer rubrum*) leaves (Li and Seeram 2018). Bamboo stem extracts have demonstrated anti-melanogenic and anti-oxidative activities in a cell-free system and B16F10 melanoma cells (Choi et al. 2018). The ethanol extract of the marula tree is very effective in boosting activities in vitro*.* ECG and EGCG in marula tree extract contribute to anti-ageing activities (Shoko et al. 2018). *Cocos nucifera* bark showed anti-oxidant and anti-depressant activities through oxidative alterations in the prefrontal cortex (Lima et al. 2016).

### UV protection activity

Extensive studies of the protective capacity of catechins against UV radiation have demonstrated that catechins are capable of enhancing the photo stability and protection of skin from UV rays. Studies have also been conducted to find effective uses for catechins in various fields, such as the prevention of skin ageing, by increasing their efficacy and stability. Catechins improve the stability of EGCG nanoethosomal suspensions to enhance the effectiveness of inhibiting UVB-induced skin damage (Zhang et al. 2016). Emulsification of catechins increases the permeation of the skin, protective capacity against UV rays, and anti-ageing effects (Yoshino et al. 2013). Various analyses, including3-(4,5-dimethylthiazol-2-yl)-2,5-diphenyltetrazolium bromide (MTT) and western blot assays, show that ECG is a powerful cure for UVB-induced damage to HaCaT keratinocytes (Huang et al. 2007). Exposure to simulated solar radiation with sunscreen sorbents showed that grape seed extracts have broad-spectrum protection due to their high photostability and a red shift over the entire UVA and UVB ray index (Martincigh and Ollengo 2016). Flavonoids show high light and heat stability in the preservation and release of methacrylic acid-grafted poly (*N*-vinyl-pyrrolidone) acid-grafted (*N*-vinyl-pyrrolidone) (Parisi et al. 2012). The inhibitory activity against mushroom tyrosinase of components isolated from *Neolitsea aciculate* demonstrates this plant could be a source of anti-melanin-producing agents (Kim et al. 2012). Cultured UV-induced human keratinocytes were treated with EGCG, and the effects on inflammatory pathways and nuclear translocation of the transcription factor NF-κB were assessed. EGCG inhibited UVB- and UVA-induced inflammatory pathways and apoptosis in cultured human keratinocytes (Xia et al. 2005).

### Anti-microbial activity

Research is underway to produce biological and functional cosmetics using the natural anti-microbial properties of catechins. Human epithelial KB cells cell experiments show thatflavan-3-ols and proanthocyanidin from *Limonium brasiliense* (*L. brasiliense*) interact with gingipains to inhibit the adhesion of *Porphyromonas gingivalis* (*P. gingivalis*) to epithelial host cells (de Oliveira et al. 2017). In studies of the anti-microbial activity of fullerene and its hydroxylated derivatives, C60 (OH)44 was as potent and broadly effective as catechin, which was used as a control for evaluation (Aoshima et al. 2009). Green tea extracts significantly reduced the levels of *Streptococcus mutans* (*S. mutans*) in saliva and dental plaques of children (Goyal et al. 2017).

### Anti-allergenic and anti-inflammatory activities

Allergies are caused by an over active immune system reaction, producing itching and inflammation. Contact with certain allergens leads to a sensitive condition. Studies have been conducted on the anti-allergenic activity of catechins. The anti-allergenic components of the oolong tea tree and the inhibitory activity of catechins on histamine released from rat peritoneal mast cells passively sensitised with the anti-egg albumin IgE antibody were investigated. GCG was the most potent anti-allergenic component among tea catechins (Ohmori et al. 1995). Extracts of *Acerola bagasse* (*A. bagasse*) can modulate the activity of proteases that act on coagulant, anti-coagulant, and thrombolytic activities as well as the destruction of phospholipids, thereby decreasing inflammation and platelet aggregation (Marques et al. 2018). Methanol extracts of the stem bark of *Vitellaria paradoxa* (*V. paradoxa*) showed anti-inflammatory and anti-arthritic activities in acute and chronic inflammation in Wistar albino rats (Foyet et al. 2015). Chlorhexidine and green tea extracts reduced dentin corrosion and wear. Some matrix metallo protease inhibitors may be a preventative measure to prevent dentin erosion-abrasion (Magalhães et al. 2009).

### Anti-viral and anti-cancer activities

Many studies have been conducted on the prevention and treatment of viral infections (measles, AIDS, chicken pox, SARS, MERS, Ebola, etc.). An experimental study demonstrated the anti-influenza virus activity of green tea catechins (Ide et al. 2014). Ent EC-(4alpha→8) EC (Ent-Epiafzelechin-(4alpha -> 8) -epiafzelechin of *Cassiajavanica* did not affect cell viability and proliferation but interfered with herpes simplex virus cell penetration and adhesion (Cheng 2006). In clinical trials, gargling with green tea three times a day did not alter the rate of contracting the influenza virus. The researchers suggested that further study of catechin anti-viral activities are needed (Ide et al. 2014). Studies have found anti-cancer substances in plants that inhibit cancer cell proliferation, including catechins. Polyphenol-rich extracts from *Lawsonia inermis* (*L. inermis*) L. (Henna) inhibit oxidative radicals and cancer cell proliferation (Kumar et al. 2016).

### Activation of skin barrier passage

Catechins have excellent anti-oxidant activity, but their high molecular weight and binding to the lipid bilayer of the skin are obstacles to passing the skin barrier. There have been numerous attempts to overcome this problem. Microneedle-mediated intradermal delivery enables EGCG to penetrate to deeper skin layers. Skin microporation with maltose microneedles facilitates the penetration of EGCG across the stratum corneum into the deeper skin layers, including the viable epidermis and dermis (Puri et al. 2016). Based on the use of oil-water emulsions with different oil contents, a mixture of polyphenols containing catechins using Franz-type diffusion cells permeated the epidermis and dermis in pig skin in vitro (Zillich et al. 2013). Hydrophilic additives reduce the activity of flavonoids by increasing their solubility. Skin penetration of flavonoids from grape leaf extract as well asrutin, quercetin, and catechins occurs through lipophilic membranes (Arct et al. 2002). EGCG, quercetin, 14-EGCG, and *Ginkgo biloba* extracts show excellent skin penetration in fresh white skin obtained from abdominal surgery on static Franz-type diffusion cells (dal Belo et al. 2009). Monoglycerol Ester (MGE)-liquid crystal (LC)-forming lipid and glycerol monoolate (GMO)-LC formulations have improved skin penetration from various physico-chemical properties of the drug. MGE formulations have lower viscosity, faster drug release, and better skin permeability than GMO formulations. The low viscosity of the MGE-LC-preparations might affect drug diffusion and permeability through the skin (Kadhum et al. 2017). Liposomes can actively pass skin layers through artificial phosphor lipid membranes. Phospholipids have an outstanding affinity for certain groups of flavonoids, and a mixture of catechins and phytosomes, a complex of naturally active components and phospholipids (mainly lecithin), enhances skin elasticity (Bombardelli 1991). The interaction between fish collagen peptide (FCP) and EGCG was analysed using spectroscopic techniques, such as fluorescence spectres copy circular dichroism and Fourier-transform infrared spectroscopy (FTIR). More exposure of proline was found when FCP-EGCG complexes formed. FCP acts as an enhancer of EGCG and increases the absorption of EGCG into the skin and the body (Yang et al. 2015c). Chitosan microparticles containing green tea extracts show permeation of catechins into subcutaneous tissues, and metabolism studies show that chitosan microparticles improve subcutaneous delivery of catechins while limiting their degradation by skin enzymes (Wisuitiprot et al. 2011).

### Promotion of cell activity

The effects of natural extracts, including catechins, on cell activity have been studied extensively. Extracts of black, green, and white tea have anti-melanogenic activities in immortalised melanocytes. Fermented tea leaves have the lowest cytotoxicity and the highest anti-melanogenic activities (Kim et al. 2015). EGCG reduced the secretion and production of melanin in human melanoma cells in a mechanistic study promoting skin hydration that measured anti-oxidant and pigmentation properties. EGCG increases hyaluronic acid synthase gene expression and cell proliferation (Kim et al. 2018). EGCG-5′-O-α-glucopyranoside (EGCG-5′Glu), an EGCG derivative, has anti-oxidative effects in both cell-free and cellular systems. EGCG-5′Glu restores reactive oxygen species (ROS)-mediated cell viability, regulates caspases and cell survival molecules, and increases cell proliferation by modulating NF-κB activity (Han et al. 2018).

### Sludge utilisation

High value-added sludge utilisation in pharmaceuticals, cosmetics, and food has made considerable progress. Tannin, an extract of solid waste produced in chestnut industrial processes, is an effective natural anti-oxidant for the cosmetic, food, and pharmaceutical industries (Aires et al. 2016). Used coffee grounds are a great source of bioactive compounds of interest to the cosmetic and pharmaceutical industries, and methylanthines and phenols are health-related compounds present in used coffee grounds. FTIR has been used to evaluate useful active ingredients in used coffee grounds (Magalhães et al. 2016). Identification and quantification of phenolic compounds and radical scavenging activities of the two by-products of *Vitis vinifera* L. cv noir showed that they have high potential as anti-oxidants (Reis et al. 2016). Procyanidins, composed of catechin oligomers, function in anti-oxidant activity, metal chelation, radical trapping, and direct enzyme binding. Based on these results, procyanidin oligomers strongly bind with permanent keratin hairs and inhibit hair destruction caused by oxidative damage (Kim 2011). Laccase catalyses the polymerisation of phenolic compounds, suggesting that laccase-catalysed polymerisation of natural phenols may be applied to the development of new cosmetic pigments (Jeon et al. 2010). In peel and seed tests, peel extracts show higher total polyphenol content and anti-oxidant activity (Kosińska et al. 2012). In characterisation and quantification analyses using HPLC-ESI-MS/MS, the highest anti-oxidant activity levels in the edible parts of araticum fruits were in the peel, followed by the pulp, and then the seeds (Arruda et al. 2017). In tests of cocoa bean husks using hot water treatment, anti-oxidant-rich extracts of phenol, sugar, and the obromine were produced when treated at 170 °C for 30 min (Hernández-Hernández et al. 2018). *Artocarpus heterophyllus* (*A. hererophyllus*) shell is a good source of natural anti-oxidants and other physiologically active substances including catechins, according to the results of various analyses, such as LC-MS/MS and GC/MS (Sharma et al. 2013). Sapucaia nuts and their by-products are rich in phenolic compounds that are high in anti-oxidant activity. The content of phenol is especially high in the shell (Demoliner et al. 2018). In another study, the anti-oxidant activities of the bark fibres of four coconut cultivars were examined, and the phenolic components and anti-oxidant activities of the coconut shells were confirmed (Oliveira et al. 2013).

### Stability

Catechins have high anti-oxidant activities and protect the skin from the sun’s UV rays. Many studies are underway to stabilise catechins, which are highly unstable in sunlight. Addition of α-lipoic acid to catechins can produce an effective anti-oxidant by stabilising EGCG (Scalia et al. 2013). Components should be closely monitored when evaluating the compatibility of catechins and excipients commonly used for micro- and nanoemulsions in complementary and thermal assays. Especially for preparations containing liposomes, heat-based production processes should be avoided (Ferreira-Nunes et al. 2018). Flavonoids, alkaloids, and phenolic acids in green tea toothpastes were analysed for stability at various pH levels and were more stable at low pH (Jang et al. 2014). The photo stability of EGCG was examined under the same conditions using a water-soluble UVB filter, benzophenone-4(BP-4). The results showed that photo stability was concentration dependent; the maximum level of EGCG photo stabilisation (catechin loss, 29.4 ± 2.2%) was attained in the presence of 2.1% (w/w) BP-4 (Bianchi et al. 2011). A study of catechin-based collagen stabilisation showed that hydrophobic interactions and hydrogen bonding interaction affected collagen stabilisation by plant polyphenols (Madhan et al. 2005). *C. decapetala* extracts have anti-oxidant properties due to the phenolic compounds in the leaves. At a concentration of 0.2%, *C. decapetala* extracts reduced the oxidative degradation of the oil-in-water emulsion (Gallego et al. 2017).

### Tissue biopsy culture model

The results of in vivo applications of catechins are not the same as in vitro results. Tissue bio culture models play a valuable role by replacing animal experiments in studies of catechins. To prove the principle that proteins and key gene markers may be altered in an optimised whole-tissue biopsy culture model, topical formulations containing green tea catechins were examined in a skin biopsy culture model (Sidgwick et al. 2016). EpiDerm has anti-oxidant properties like those of living organisms and can eliminate oxidative stress factors caused by EGCG under in vitro experimental conditions (Yuki et al. 2013). In an experiment using HaCaT and RBL-2H3 cells, the safety and anti-inflammatory effect of nanoencapsulated lipid-soluble green tea leaf extracts using the supercritical CO_2_ extraction method were objectively proven (Shin et al. 2019).

### Safety for human application

Although natural extracts are effective as anti-oxidants and anti-microbials, the safety of catechins should be ensured in actual human applications. Studies on the safety of catechins have been conducted in animal experiments and human clinical tests. Propionidin B-2 [EC- (4beta→8)-EC] promotes hair growth, and safety studies for human application are needed. Local procyanidin B-2 was safe and acceptable in a series of toxicity tests. Mutagenicity tests using guinea pigs, bacteria, and rabbits show that procyanidin B-2 is not a mutagen (Takahashi et al. 1999). In characterisation and biocompatibility studies of “green” silver nanoparticles using green tea polyphenols, silver nanoparticles were nontoxic and biocompatible (Moulton et al. 2010).

### Anti-oxidant properties of catechins used for other applications

In addition to their direct anti-oxidant activities, catechins are being studied to increase their utility in various fields. To increase the rate of the hair dying process using plant products, phenol-derived polymeric dyes from *Trametes versicolor* use a laccase reaction with catechins and catechol to achieve a permanent keratin hair dye of various colours and shades (Im and Jeon 2016). Food packaging materials or active membranes containing anti-oxidants, such as catechin-derived EGCG and EC, are a new way to reduce the oxidation of foods, cosmetics, and pharmaceuticals using biopolymer materials. The anti-oxidative activity of the film was measured by the removal of methanol extracts containing catechins and EC, and their amounts were 32.90% and 36.68%, respectively (Iñiguez-Franco et al. 2012). Tannic acid, EGCG, and ECG were bound to collagen by extensive hydrogen bonding augmented by hydrophobic interactions. They prevented the free access of collagenase to the active areas in collagen chains (Jackson et al. 2010).

### Synergistic effects by the method and process of extraction

Many attempts have been made to improve the effectiveness and utilisation of catechins and to efficiently apply their anti-oxidant properties to the human body. Anti-oxidative and UV-barrier properties of the molecules can be used for cosmetic and dermatological formulations after a selective high-performance liquid chromatography (HPLC) method is developed and verified for evaluating the optimal efficacy of catechins in the development of topical formulations (Ferreira-Nunes et al. 2017). Near-infrared spectroscopy (NIRS) has been proposed as a rapid and non-destructive way to measure the contents of three main phenolics (caffeic acid, (+)-catechin, and chlorogenic acid) (Magalhães et al. 2016). Chemical modification of anthocyanin and procyanidins to more lipophilic compounds by mass spectroscopy has the advantage of increasing bioavailability in biological matrices because anti-oxidation activity increases based on the acylation of procyanidin B4 by saturated fatty acids (Cruz et al. 2015). Polyphenols and collagen peptides can be applied to the design of clear products, via the formation of lactoferrin (LF)–EGCG aggregates, which are destroyed chiefly through competition mechanisms with EGCG molecules (Yang et al. 2015a). The mechanism and structural properties of trivalent aggregates of LF and pectin in a multispectral analysis show that the fluorescence intensity of LF decreases while that of EGCG increases (Yang et al. 2015b). FTIR spectral analysis confirmed that the hydrogen bonds between the aliphatic, catechin, and aromatic hydroxyl groups on gelatin were responsible for the self-assembly of nanoparticles. In free-radical experiments, catechins could be protected by nanoparticles and last for an extended period (Chen et al. 2010). An efficient, precise, and reliable method was developed to quantify polyphenol catechins and EC in aguaraná extract solution using an HPLC-PDA method (Klein et al. 2012). Three different solvents and two extraction methods were used to compare the total polyphenol and flavonoid contents of tara pod extracts. The total polyphenol content was highest when a 75% ethanol solution was used in an hour-long ultrasonic process, and the flavonoid content was highest when it was extracted for 24 h in cold water. However, water extracts were effective only in the early stages of the oxidation process, showing that 75% ethanol extraction is the best method for polyphenol isolation (Skowyra et al. 2013). Asynergistic study between process parameters found that augmentation of the ultrasonic treatment process significantly speeds the recovery of phenolic anti-oxidants and reduces the processing time (Arruda et al. 2019). Procyanidin extracts of grape seeds prevent damage to most tissues and molecules from nanoparticle treatment (Niu et al. 2017).

## Conclusions

Table 1 summarises the activities of catechins and their applications. Catechins are used as materials to promote health, to prevent and treat diseases, and for cosmetic purposes. Studies of their high anti-oxidant activities found in plants and their by-products are continually being conducted. Extensive studies have been conducted on the UV protective activities of catechins to enhance their photo stability, efficacy, and stability for their use in various fields, including slowing the skin-aging process. EGCG nanoparticles inhibit UVA damage, grape seeds have photo stability against UVA rays, and catechins inhibit UVA- and UVB-induced inflammatory pathways. Anti-microbial activities of catechins were shown to inhibit the adhesion of *P. gingivalis* to host epithelial cells, as flavan-3-ols and proanthocyanidins of *L. brasiliense* interact with gingipains. Green tea extracts significantly reduce the levels of *S*. *mutans* in saliva as well as dental plaques. GCG is the most potent anti-allergenic component among all tea catechins. Extracts of *A. bagasse* coagulants reduce inflammation and platelet aggregation. Extracts of *V. paradoxa* stem barks have anti-inflammatory and anti-arthritic properties. Green tea catechins have anti-influenza properties and have been shown to inhibit herpes simplex enzyme cell penetration and adhesion. Polyphenol-rich W-LI extracts from *L. inermis* (Henna) can inhibit oxidative radicals and cancer cell proliferation. Nanoparticles delivered through microneedles into human skin and oil-water emulsions with different oil contents enhance skin penetration and retention using Franz-type diffusion cells. The lipophilic membrane model increases rutin and quercetin contents, including flavonoids, and enhances skin penetration and retention due to the interaction of fish collagen and EGCG. FCP acts as an enhancer of catechins and increases absorption of catechins into the skin and the body, and chitosan microparticles improve the transdermal delivery of catechins. Catechin promotes cellular activities, and tea extracts inhibit melanin production. Fermented tea has the highest anti-melanogenic activity and the lowest cytotoxicity. Hyaluronic acid synthase reduces melanin secretion, EGCG derivatives restore ROS-mediated cell viability, and EGCG-5′Glu increases cell proliferation. Many studies have been conducted to obtain anti-oxidants from sludge, including chestnut shells, coffee grounds, *A. heterophyllus* shells, and coconut shells, with results that show good anti-oxidant activity. The use of sludge has been studied widely due to its high utility value, environmental protection, and interest in up-cycling products.

**Table 1:** Activities of catechins and their applications

Catechin activity	Application	References
Anti-oxidant activities	Maximising the efficacy of anti-oxidants and catechins in new substances	D’Urso et al. 2018, Gallego et al. 2017, Spizzirri et al. 2009, Nadim et al. 2014, Feng et al. 2014, Feng et al. 2013, Zhong and Shahidi 2011, Muhammad et al. 2014, Li and Seeram 2018, Choi et al. 2018, Shoko et al. 2018, Lima et al. 2016
UV protection	Improve the stability of catechins in sunlight and increase the UV protection effect	Zhang et al. 2016, Yoshino et al. 2013, Niu et al. 2017, Huang et al. 2007, Martincigh and Ollengo 2016, Parisi et al. 2012, Kim et al. 2012, Xia et al. 2005
Anti-microbial activities	Demonstrates anti-microbial activity and develops pharmaceuticals and functional cosmetics	de Oliveira et al. 2017, Aoshima et al. 2009, Goyal et al. 2017
Anti-allergenic and anti-inflammatory activities	Has an anti-allergenic component and anti-inflammatory and anti-arthritic activity	Ohmori et al. 1995, Marques et al. 2018, Foyet et al. 2015, Magalhães et al. 2009
Anti-viral activities	Shows anti-influenza activity and interferes with cell infiltration and attachment of herpes simplex virus	Ide et al. 2014, Cheng 2006
Anti-cancer activities	Extract from *Lawsonia inermis* (Henna) can inhibit proliferation of cancer cells	Kumar et al. 2016
Activation of skin barrier passage	Various methods have enhanced the skin penetration of epigallocatechin-3-gallate (EGCG) anti-oxidants	Puri et al. 2016, Zillich et al. 2013, dal Belo et al. 2009, Kadhum et al. 2017, Bombardelli 1991, Arct et al. 2002, Yang et al. 2015c, Wisuitiprot et al. 2011
Promoting cell activities	Effective for low cytotoxicity and anti-melanin production and improves reactive oxygen species (ROS)-mediated cell viability and cell proliferation	Kim et al. 2015, Kim et al. 2018
Sludge utilisation	Anti-oxidant components were extracted from various sludge, suggesting that they could be developed into foods, cosmetics, and pharmaceuticals	Aires et al. 2016, Magalhães et al. 2015, Reis et al. 2016, Kosińska et al. 2012, Arruda et al. 2017, Hernández-Hernández et al. 2018, Sharma et al. 2013, Demoliner et al. 2018, Oliveira et al. 2013
Stability	Aspects exploited for improving stability include sunlight, oxidation, compound stability, and collagen stabilisation	Scalia et al. 2013, Ferreira-Nunes et al. 2018, Jang et al. 2014, Bianchi et al. 2011, Madhan et al. 2005, Gallego et al. 2017
	Relatively stable at low pH and the extraction efficiency of anti-oxidants was high	Tsuchiya et al. 1997, Jang et al. 2014
Tissue biopsy culture model	Demonstrated efficacy in vivo and vitro culture models	Sidgwick et al. 2016, Ow and Stupans 2003, Yuki et al. 2013, Moulton et al. 2010
Safety for human applications	In vivo experiments demonstrate safety	Takahashi et al. 1999, Moulton et al. 2010
Anti-oxidant properties of catechins used for other applications	Dyes, packaging materials, nanoparticles, and biocompatibility	Im and Jeon 2016, Iñiguez-Franco et al. 2012, Kim 2011, Jeon et al. 2009, Rojas et al. 2005, Jackson et al. 2010
Synergistic effect by extraction method and process	Chemical modification, molecular interaction mechanisms, hydrogen bonding, and nanoparticle treatment increase efficiency	Ferreira-Nunes et al. 2017, Magalhães et al. 2016, Cruz et al. 2015, Yang et al. 2015a, Chen et al. 2010, Yang et al. 2015b, Niu et al. 2017, Klein et al. 2012, Skowyra et al. 2013, Arruda et al. 2019

Catechins are highly unstable in sunlight, and research is underway to stabilise catechins. Addition of α-lipoic acid to catechins is effective for its stabilisation. BP-4, a soluble UVB filter, can stabilise EGCG to produce effective anti-oxidants. Catechin preparations made with lipid ingredients are less stable in heat, so it is necessary to avoid heating them as much as possible. The lower the pH level, the more stable the anti-oxidant activities of green tea compounds. This suggests that more research is required on the effect of pH on the various activities of catechins.

Catechins have been used in the tissue biopsy culture model to achieve optimised effects like those in an in vivo application. The anti-oxidant properties of EpiDerm are like those of living organisms, and the stability and anti-inflammatory effects of catechins in HaCaT cells and RBL-2H3 cells were objectively proven. In safety tests for human applications, propionidinB-2 (epicatechin) was nontoxic and nonmutagenic. The anti-oxidant properties of catechins make them suitable for use in hair dyes and containers for medicines and cosmetics to reduce oxidation of the contents. HPLC, NIRS, nanoparticles, cold water, 75% ethanol solution, and ultrasonic treatment are proposed methods to increase human body applications and the extraction efficiency of catechins. All these studies and achievements suggest that the anti-oxidant activities of catechins will contribute significantly to the development of cosmetics and to human health.

## Data Availability

Data sharing not applicable to this article as no datasets were generated or analyzed during the current study.

## Abbreviations

BP-4: Benzophenone-4
C: ((-)-Catechin)
CG: ((-)-Catechingallate)
EC: ((-)-Epicatechin)
ECG: ((-)-Epicatechin gallate)
EGC: ((-)-Epigallocatechin)
EGCG: ((-)-Epigallocatechin gallate)
EGCG-5′Glu: EGCG-5′-O-α-glucopyranoside
FCP: Fish collagen peptide
FTIR: Fourier-transform infrared spectroscopy
GC: ((-)-Gallocatechin)
GCG: ((-)-Gallocatechingallate)
HPLC: High-performance liquid chromatography
LF: Lactoferrin
NIRS: Near-infrared spectroscopy
ROS: Reactive oxygen species

## References

[CR1] Aires A, Carvalho R, Saavedra MJ (2016). Valorization of solid wastes from chestnut industry processing: extraction and optimization of polyphenols, tannins and ellagitannins and its potential for adhesives, cosmetic and pharmaceutical industry. Waste Manag.

[CR2] Aoshima H, Kokubo K, Shirakawa S, Ito M, Yamana S, Oshima T (2009). Antimicrobial activity of fullerenes and their hydroxylated derivatives. Biocontrol Sci.

[CR3] Arct J, Bielenda B, Oborska A, Pytkowska K (2003). The tea and its cosmetic application. J Appl Cosmetol.

[CR4] Arct J, Oborska A, Mojski M, Binkowska A, Swidzikowska B (2002). Common cosmetic hydrophilic ingredients as penetration modifiers of flavonoids. Int J Cosmet Sci.

[CR5] Arruda HS, Pereira GA, de Morais DR, Eberlin MN, Pastore GM (2018). Determination of free, esterified, glycosylated and insoluble-bound phenolics composition in the edible part of araticum fruit (*Annona crassiflora* Mart.) and its by-products by HPLC-ESI-MS/MS. Food Chem.

[CR6] Arruda HS, Silva EK, Pereira GA, Angolini CFF, Eberlin MN, Meireles MAA (2019). Effects of high-intensity ultrasound process parameters on the phenolic compounds recovery from araticum peel. Ultrason Sonochem.

[CR7] Bianchi A, Marchetti N, Scalia S (2011). Photodegradation of (−)-epigallocatechin-3-gallate in topical cream formulations and its photostabilization. J Pharm Biomed Anal.

[CR8] Bombardelli E (1991). Phytosome : new cosmetic delivery system. Boll Chim Farm.

[CR9] Chen Y-C, Yu S-H, Tsai G-J, Tang D-W, Mi F-L, Peng Y-P (2010). Novel technology for the preparation of self-assembled catechin/gelatin nanoparticles and their characterization. J Agric Food Chem.

[CR10] Cheng H-Y (2006). ent-Epiafzelechin-(4 ->8)-epiafzelechin extracted from *Cassia javanica* inhibits herpes simplex virus type 2 replication. J Med Microbiol.

[CR11] Choi M-H, Jo H-G, Yang J, Ki S, Shin H-J (2018). Antioxidative and anti-melanogenic activities of bamboo stems (*Phyllostachys nigra* variety henosis) via PKA/CREB-mediated MITF downregulation in B16F10 melanoma cells. Int J Mol Sci.

[CR12] Cruz L, Fernandes VC, Araújo P, Mateus N, de Freitas V (2015). Synthesis, characterisation and antioxidant features of procyanidin B4 and malvidin-3-glucoside stearic acid derivatives. Food Chem.

[CR13] D’Urso G, Pizza C, Piacente S, Montoro P (2018). Combination of LC–MS based metabolomics and antioxidant activity for evaluation of bioactive compounds in *Fragaria vesca* leaves from Italy. J Pharm Biomed Anal.

[CR14] dal Belo SE, Gaspar LR, PMBG MC, Marty J-P (2009). Skin penetration of epigallocatechin-3-gallate and quercetin from green tea and “*Ginkgo biloba*” extracts vehiculated in cosmetic formulations. Skin Pharmacol Physiol.

[CR15] de Oliveira CA, Hensel A, Mello JCP, Pinha AB, Panizzon GP, Lechtenberg M (2017). Flavan-3-ols and proanthocyanidins from *Limonium brasiliense* inhibit the adhesion of *Porphyromonas gingivalis* to epithelial host cells by interaction with gingipains. Fitoterapia.

[CR16] Demoliner F, de BrittoPolicarpi P, Vasconcelos LFL, Vitali L, Micke GA, Block JM (2018). Sapucaia nut (*Lecythis pisonis* Cambess) and its by-products: a promising and underutilized source of bioactive compounds. Part II: phenolic compounds profile. Food Res Int.

[CR17] Feng B, Fang Y, Wei SM (2013). Effect and mechanism of epigallocatechin-3-gallate (EGCG) against the hydrogen peroxide-induced oxidative damage in human dermal fibroblasts. J Cosmet Sci.

[CR18] Feng H-L, Tian L, Chai W-M, Chen X-X, Shi Y, Gao Y-S (2014). Isolation and purification of condensed tannins from flamboyant tree and their antioxidant and antityrosinase activities. Appl Biochem Biotechnol.

[CR19] Ferreira-Nunes R, Angelo T, da Silva SMM, Magalhães PO, Gratieri T, da Cunha-Filho MSS (2017). Versatile chromatographic method for catechin determination in development of topical formulations containing natural extracts. Biomed Chromatogr.

[CR20] Ferreira-Nunes R, Gratieri T, Gelfuso GM, Cunha-Filho M (2018). Mixture design applied in compatibility studies of catechin and lipid compounds. J Pharm Biomed Anal.

[CR21] Foyet H, Tsala D, ZogoEssono Bodo J, Carine A, Heroyne L, Oben E (2015). Anti-inflammatory and anti-arthritic activity of a methanol extract from *Vitellaria paradoxa* stem bark. Pharm Res.

[CR22] Fung S-T, Ho CK, Choi S-W, Chung W-Y, Benzie IFF (2012). Comparison of catechin profiles in human plasma and urine after single dosing and regular intake of green tea (*Camellia sinensis*). Br J Nutr.

[CR23] Gallego M, Skowyra M, Gordon M, Azman N, Almajano M (2017). Effect of leaves of *Caesalpinia decapetala* on oxidative stability of oil-in-water emulsions. Antioxidants.

[CR24] Goyal A, Bhat M, Sharma M, Garg M, Khairwa A, Garg R (2017). Effect of green tea mouth rinse on *Streptococcus mutans* in plaque and saliva in children: an *in vivo* study. J Indian Soc Pedod Prev Dent.

[CR25] Gulati A, Rajkumar S, Karthigeyan S, Sud RK, Vijayan D, Thomas J (2009). Catechin and catechin fractions as biochemical markers to study the diversity of Indian tea (*Camellia sinensis* (L.) O. Kuntze) germplasm. Chem Biodivers.

[CR26] Han S, Kim E, Hwang K, Ratan Z, Hwang H, Kim E-M (2018). Cytoprotective effect of epigallocatechin gallate (EGCG)-5′-O-α-glucopyranoside, a novel EGCG derivative. Int J Mol Sci.

[CR27] Hernández-Hernández C, Morales-Sillero A, Fernández-Bolaños J, Bermúdez-Oria A, Morales AA, Rodríguez-Gutiérrez G (2018). Cocoa bean husk: industrial source of antioxidant phenolic extract. J Sci Food Agric.

[CR28] Huang CC, Wu WB, Fang JY, Chiang HS, Chen SK, Chen BH, Chen YT, Hung CF (2007). (−)-Epicatechin-3-gallate, a green tea polyphenol is a potent agent against UVB-induced damage in HaCaT keratinocytes. Molecules..

[CR29] Ide K, Yamada H, Matsushita K, Ito M, Nojiri K, Toyoizumi K (2014). Effects of green tea gargling on the prevention of influenza infection in high school students: a randomized controlled study. PLoS One.

[CR30] Im KM, Jeon J-R (2016). Synthesis of plant phenol-derived polymeric dyes for direct or mordant-based hair dyeing. J Vis Exp.

[CR31] Iñiguez-Franco F, Soto-Valdez H, Peralta E, Ayala-Zavala JF, Auras R, Gámez-Meza N (2012). Antioxidant activity and diffusion of catechin and epicatechin from antioxidant active films made of poly (l-lactic acid). J Agric Food Chem.

[CR32] Jackson JK, Zhao J, Wong W, Burt HM (2010). The inhibition of collagenase induced degradation of collagen by the galloyl-containing polyphenols tannic acid, epigallocatechin gallate and epicatechingallate. J Mater Sci Mater Med.

[CR33] Jang J-H, Park Y-D, Ahn H-K, Kim S-J, Lee J-Y, Kim E-C (2014). Analysis of green tea compounds and their stability in dentifrices of different pH levels. Chem Pharm Bull.

[CR34] Jeon J-R, Kim E-J, Murugesan K, Park H-K, Kim Y-M, Kwon J-H (2010). Laccase-catalysed polymeric dye synthesis from plant-derived phenols for potential application in hair dyeing: enzymatic colourations driven by homo- or hetero-polymer synthesis. Microb Biotechnol.

[CR35] Jin Y, Jin CH, Ho RK (2006). Separation of catechin compounds from different teas. Biotechnol J.

[CR36] Kadhum WR, Sekiguchi S, Hijikuro I, Todo H, Sugibayashi K (2017). A novel chemical enhancer approach for transdermal drug delivery with C17-monoglycerol ester liquid crystal-forming lipid. J Oleo Sci.

[CR37] Kim E, Hwang K, Lee J, Han S, Kim E-M, Park J (2018). Skin protective effect of epigallocatechin gallate. Int J Mol Sci.

[CR38] Kim M-M (2011). Effect of procyandin oligomers on oxidative hair damage. Skin Res Technol.

[CR39] Kim SS, Hyun C-G, Choi YH, Lee NH (2012). Tyrosinase inhibitory activities of the compounds isolated from *Neolitsea aciculata* (Blume) Koidz. J Enzyme Inhib Med Chem.

[CR40] Kim YC, Choi SY, Park EY (2015). Anti-melanogenic effects of black, green, and white tea extracts on immortalized melanocytes. J Vet Sci.

[CR41] Klein T, Longhini R, de Mello JCP (2012). Development of an analytical method using reversed-phase HPLC-PDA for a semipurified extract of *Paullinia cupana* var. *sorbilis* (guaraná). Talanta.

[CR42] Kosińska A, Karamać M, Estrella I, Hernández T, Bartolomé B, Dykes GA (2012). Phenolic compound profiles and antioxidant capacity of *Persea americana* mill. Peels and seeds of two varieties. J Agric Food Chem.

[CR43] Kumar M, Chandel M, Kaur P, Pandit K, Kaur V, Kaur S (2016). Chemical composition and inhibitory effects of water extract of Henna leaves on reactive oxygen species, DNA scission and proliferation of cancer cells. EXCLI J.

[CR44] Li C, Seeram NP (2018). Ultra-fast liquid chromatography coupled with electrospray ionization time-of-flight mass spectrometry for the rapid phenolic profiling of red maple (*Acer rubrum*) leaves. J Sep Sci.

[CR45] Lima EBC, de Sousa CNS, Vasconcelos GS, Meneses LN, YF e SP, Ximenes NC (2016). Antidepressant, antioxidant and neurotrophic properties of the standardized extract of *Cocos nucifera* husk fiber in mice. J Nat Med.

[CR46] Madhan B, Subramanian V, Rao JR, Nair BU, Ramasami T (2005). Stabilization of collagen using plant polyphenol: role of catechin. Int J Biol Macromol.

[CR47] Magalhães AC, Wiegand A, Rios D, Hannas A, Attin T, Buzalaf MAR (2009). Chlorhexidine and green tea extract reduce dentin erosion and abrasion in situ. J Dent.

[CR48] Magalhães LM, Machado S, Segundo MA, Lopes JA, Páscoa RNMJ (2016). Rapid assessment of bioactive phenolics and methylxanthines in spent coffee grounds by FT-NIR spectroscopy. Talanta.

[CR49] Marques TR, Cesar PHS, Braga MA, Marcussi S, Corrêa AD (2018). Fruit bagasse phytochemicals from *Malpighia emarginata* rich in enzymatic inhibitor with modulatory action on hemostatic processes. J Food Sci.

[CR50] Martincigh BS, Ollengo MA (2016). The Photostabilizing effect of grape seed extract on three common sunscreen absorbers. Photochem Photobiol.

[CR51] Matsubara T, Wataoka I, Urakawa H, Yasunaga H (2013). Effect of reaction pH and CuSO_4_ addition on the formation of catechinone due to oxidation of (+)-catechin. Int J Cosmet Sci.

[CR52] Moulton MC, Braydich-Stolle LK, Nadagouda MN, Kunzelman S, Hussain SM, Varma RS (2010). Synthesis, characterization and biocompatibility of “green” synthesized silver nanoparticles using tea polyphenols. Nanoscale.

[CR53] Muhammad D, Hubert J, Lalun N, Renault J-H, Bobichon H, Nour M (2014). Isolation of flavonoids and triterpenoids from the fruits of *Alphitonia neocaledonica* and evaluation of their anti-oxidant, anti-tyrosinase and cytotoxic activities. Phytochem Anal.

[CR54] Nadim M, Auriol D, Lamerant-FayeL N, Lefèvre F, Dubanet L, Redziniak G (2014). Improvement of polyphenol properties upon glucosylation in a UV-induced skin cell ageing model. Int J Cosmet Sci.

[CR55] Niu L, Shao M, Liu Y, Hu J, Li R, Xie H (2017). Reduction of oxidative damages induced by titanium dioxide nanoparticles correlates with induction of the Nrf2 pathway by GSPE supplementation in mice. Chem Biol Interact.

[CR56] Ohmori Y, Ito M, Kishi M, Mizutani H, Katada T, Konishi H (1995). Antiallergic constituents from oolong tea stem. Biol Pharm Bull.

[CR57] Oliveira MB, Valentim IB, de Vasconcelos CC, Omena CM, Bechara EJ, da Costa JG, Freitas Mde L, Sant'Ana AE, Goulart MO (2013). *Cocos nucifera* Linn. (Palmae) husk fiber ethanolic extract: antioxidant capacity and electrochemical investigation. Comb Chem High Throughput Screen.

[CR58] Ow Y-Y, Stupans I (2003). Gallic acid and gallic acid derivatives: effects on drug metabolizing enzymes. Curr Drug Metab.

[CR59] Parisi OI, Puoci F, Iemma F, Curcio M, Cirillo G, Spizzirri UG (2012). Flavonoids preservation and release by methacrylic acid-grafted (N-vinyl-pyrrolidone). Pharm Dev Technol.

[CR60] Puri A, Nguyen HX, Banga AK (2016). Microneedle-mediated intradermal delivery of epigallocatechin-3-gallate. Int J Cosmet Sci.

[CR61] Reis GM, Faccin H, Viana C, da Rosa MB, de Carvalho LM (2016). *Vitis vinifera* L. cv Pinot noir pomace and lees as potential sources of bioactive compounds. Int J Food Sci Nutr.

[CR62] Rojas LB, Quideau S, Pardon P, Charrouf Z (2005). Colorimetric evaluation of phenolic content and GC-MS characterization of phenolic composition of alimentary and cosmetic argan oil and press cake. J Agric Food Chem.

[CR63] Scalia S, Marchetti N, Bianchi A (2013). Comparative evaluation of different co-antioxidants on the photochemical- and functional-stability of epigallocatechin-3-gallate in topical creams exposed to simulated sunlight. Molecules.

[CR64] Sharma A, Gupta P, Verma AK (2013). Preliminary nutritional and biological potential of *Artocarpus heterophyllus* L. shell powder. J Food Sci Technol.

[CR65] Shi M, Nie Y, Zheng X-Q, Lu J-L, Liang Y-R, Ye J-H (2016). Ultraviolet B (UVB) Photosensitivities of tea catechins and the relevant chemical conversions. Molecules.

[CR66] Shin MC, Park SK, Jung SH (2019). The inhibitory effect on cytotoxicity and nitric oxide (NO) of the nano-encapsulated extraction of lipid-soluble green tea leaves. J Nanosci Nanotechnol.

[CR67] Shoko T, Maharaj VJ, Naidoo D, Tselanyane M, Nthambeleni R, Khorombi E (2018). Anti-aging potential of extracts from *Sclerocarya birrea* (A. Rich.) Hochst and its chemical profiling by UPLC-Q-TOF-MS. BMC Complement Altern Med.

[CR68] Sidgwick GP, McGeorge D, Bayat A (2016). Functional testing of topical skin formulations using an optimised ex vivo skin organ culture model. Arch Dermatol Res.

[CR69] Singh BN, Shankar S, Srivastava RK (2011). Green tea catechin, epigallocatechin-3-gallate (EGCG): mechanisms, perspectives and clinical applications. Biochem Pharmacol.

[CR70] Skowyra M, Falguera V, Gallego G, Peiró S, Almajano MP (2013). Antioxidant properties of aqueous and ethanolic extracts of tara (*Caesalpinia spinosa*) pods *in vitro* and in model food emulsions. J Sci Food Agric.

[CR71] Spizzirri UG, Iemma F, Puoci F, Cirillo G, Curcio M, Parisi OI (2009). Synthesis of antioxidant polymers by grafting of gallic acid and catechin on gelatin. Biomacromolecules.

[CR72] Takahashi T, Yokoo Y, Inoue T, Ishii A (1999). Toxicological studies on procyanidin B-2 for external application as a hair growing agent. Food Chem Toxicol.

[CR73] Tsuchiya H, Sato M, Kato H, Okubo T, Juneja LR, Kim M (1997). Simultaneous determination of catechins in human saliva by high-performance liquid chromatography. J Chromatogr B Biomed Sci Appl.

[CR74] Wisuitiprot W, Somsiri A, Ingkaninan K, Waranuch N (2011). In vitro human skin permeation and cutaneous metabolism of catechins from green tea extract and green tea extract-loaded chitosan microparticles. Int J Cosmet Sci.

[CR75] Xia J, Song X, Bi Z, Chu W, Wan Y (2005). UV-induced NF-κB activation and expression of IL-6 is attenuated by (-)-epigallocatechin-3-gallate in cultured human keratinocytes *in vitro*. Int J Mol Med.

[CR76] Yang W, Liu F, Xu C, Sun C, Yuan F, Gao Y (2015). Inhibition of the aggregation of lactoferrin and (−)-epigallocatechin gallate in the presence of polyphenols, oligosaccharides, and collagen peptide. J Agric Food Chem.

[CR77] Yang W, Xu C, Liu F, Sun C, Yuan F, Gao Y (2015). Fabrication mechanism and structural characteristics of the ternary aggregates by lactoferrin, pectin, and (−)-epigallocatechin gallate using multispectroscopic methods. J Agric Food Chem.

[CR78] Yang W, Yuan F, Gao YX (2015). Interaction of fish collagen peptide with epigallocatechin gallate. Guang Pu Xue Yu Guang Pu Fen Xi.

[CR79] Yoshino S, Mitoma T, Tsuruta K, Todo H, Sugibayashi K (2013). Effect of emulsification on the skin permeation and UV protection of catechin. Pharm Dev Technol.

[CR80] Yuki K, Ikeda N, Nishiyama N, Kasamatsu T (2013). The reconstructed skin micronucleus assay in EpiDerm™: reduction of false-positive results – a mechanistic study with epigallocatechin gallate. Mutat Res Genet Toxicol Environ Mutagen.

[CR81] Zhang W, Yang Y, Lv T, Fan Z, Xu Y, Yin J (2016). Sucrose esters improve the colloidal stability of nanoethosomal suspensions of (−)-epigallocatechin gallate for enhancing the effectiveness against UVB-induced skin damage. J Biomed Mater Res B Appl Biomater.

[CR82] Zhong Y, Shahidi F (2011). Lipophilized epigallocatechin gallate (EGCG) derivatives as novel antioxidants. J Agric Food Chem.

[CR83] Zillich OV, Schweiggert-Weisz U, Eisner P, Kerscher M (2015). Polyphenols as active ingredients for cosmetic products. Int J Cosmet Sci.

[CR84] Zillich OV, Schweiggert-Weisz U, Hasenkopf K, Eisner P, Kerscher M (2013). Release and *in vitro* skin permeation of polyphenols from cosmetic emulsions. Int J Cosmet Sci.

